# Breast metastasis from a renal cell cancer

**DOI:** 10.1186/1477-7819-5-25

**Published:** 2007-03-02

**Authors:** Ahmed Alzaraa, Aleksandar Vodovnik, Hugh Montgomery, Mohammed Saeed, Narinder Sharma

**Affiliations:** 1 Department of General Surgery, Calderdale Royal Hospital, Halifax, UK; 2 Department of Radiology, Calderdale Royal Hospital, Halifax, UK; 3 Department of Histopathology, Calderdale Royal Hospital, Halifax, UK

## Abstract

**Background:**

Metastases to the breast from extramammary tumours are uncommon, and metastatic renal cell carcinoma to the breast is extremely rare. We report a metastasis to the breast from a renal primary with the radiological and histopathological features.

**Case presentation:**

An 81-year-old lady was seen in the breast clinic for a right breast mass after sustaining a fall. Clinical examination and investigations revealed a metastatic cancer from a renal primary. She received surgical treatment only and is under regular follow-up in the oncology clinic.

**Conclusion:**

The treatment strategy for metastatic breast diseases is based on a proper assessment of such cases by surgeons, radiologists and histopathologists.

## Background

Metastases to the breast from extramammary tumours are uncommon, and metastatic renal cell carcinoma to the breast is extremely rare [1]. We report a metastasis to the breast from a renal primary with the radiological and histopathological features.

## Case presentation

An 81 years old lady had a right radical nephrectomy in 1999 for conventional renal cell cancer (RCC). She was discharged from the urology and oncology clinics in 2004 after 5 1/2 years follow-up with no signs of local or regional recurrence.

In December 2004, she noticed a lump in the right breast after sustaining a fall. She was referred to the breast clinic in July 2005 for further assessment.

Clinically, she had a mass in the upper outer quadrant of the right breast. The left breast was normal and there was no axillary lymphadenopathy. Abdominal examination was normal.

**Figure 1:**
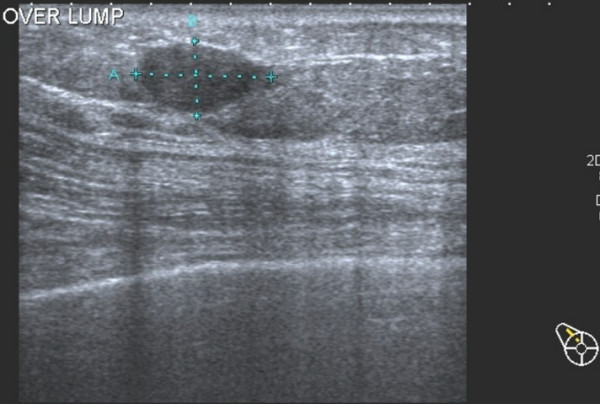
Ultrasonography showing hypoechoic mass in the right breast.

Radiology confirmed a 17 × 13 × 9 mm well circumscribed hypoechoic mass in the right upper quadrant of the right breast (Figure 1). The mass was core biopsied.

**Figure 2:**
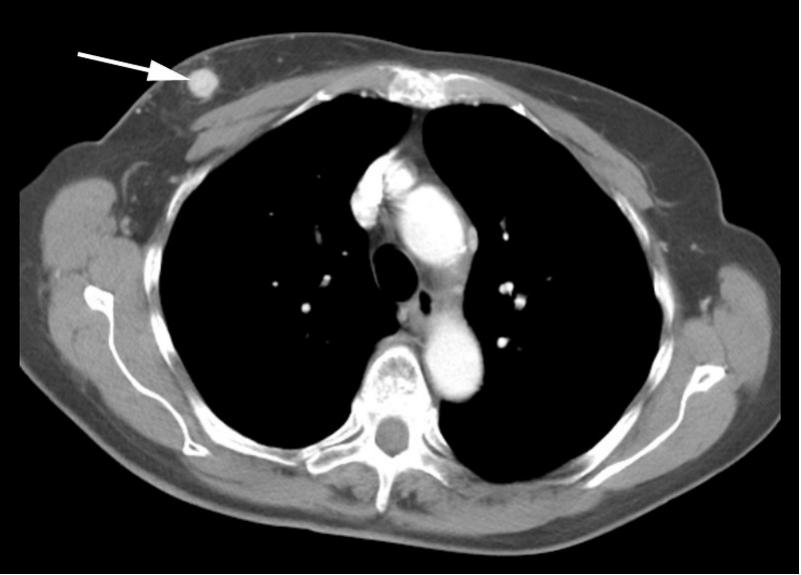
Computed tomography showing well-defined, round mass in the right breast (arrow).

**Figure 3:**
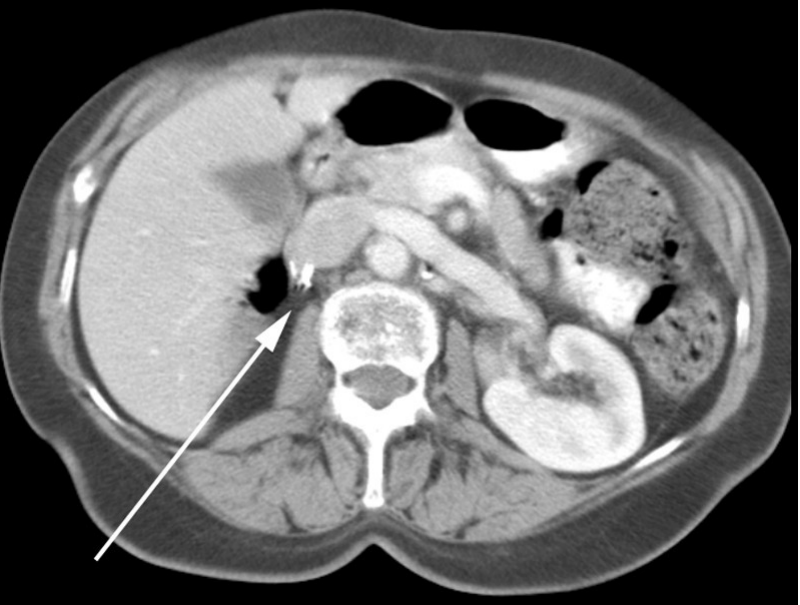
Computed tomography showing tumour mass in the renal bed (arrow points to surgical clips).

The histopathological examination revealed tumour growth consistent with conventional renal cell carcinoma. Tumour cells strongly expressed vimentin. CT scan of the chest and abdomen showed a 12 mm mass in the right breast and a 2.7 cm metastatic deposit at the right renal bed (Figures 2 and 3). The lungs and the liver were normal. The lump was excised in October 2005.

**Figure 4:**
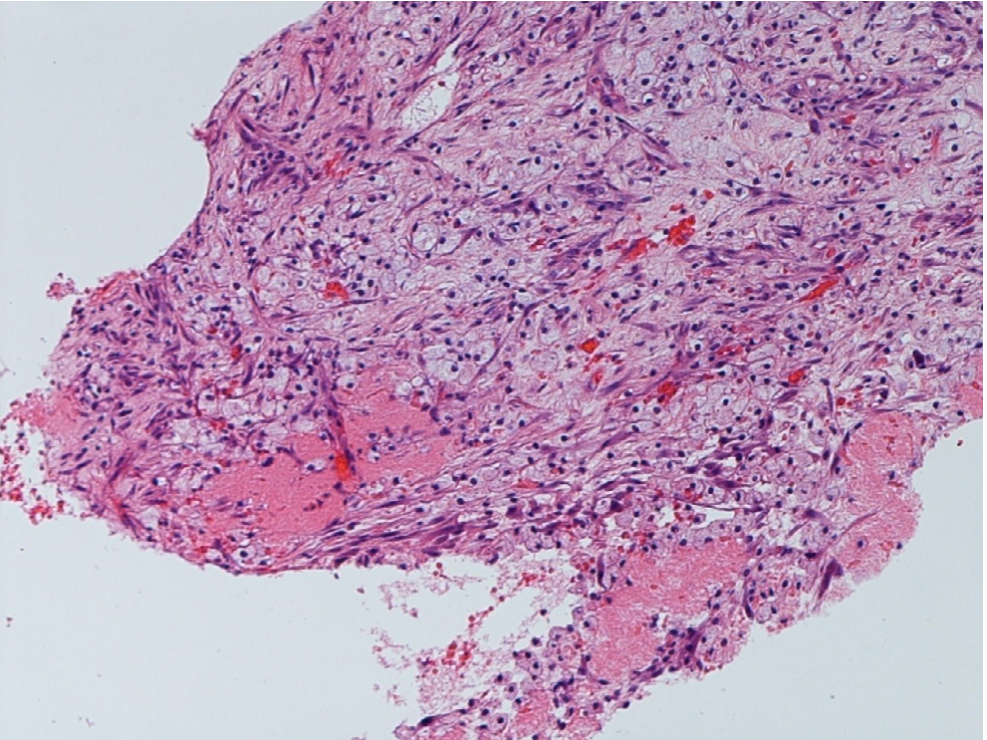
Photomicrograph showing islands of tumour cells with clear cytoplasm, lying in fibrovascular stroma (Hematoxylin & Eosin, 10×).

The gross examination of the specimen confirmed metastasis from a renal primary (Figure 4). There was no evidence of *in situ *ductal or lobular disease.

The patient was offered Interferon treatment, but she preferred to hold on therapy as an alternative. She is under regular follow-up in the oncology clinic.

## Discussion

Metastases to the breast from extramammary primary malignancies are rare. The incidence of metastasis to the breast in various clinical autopsy studies ranges from 5%–6.6% [3]. Both breasts are equally affected and bilateral involvement is not uncommon. Solitary discrete lesions occur in 85% [4].

The primitive neoplasms that most frequently metastasise to the breast are malignant melanoma, lymphoma, lung cancer and in men, prostatic cancer [1-3]. Breast metastasis from a renal tumour is extremely rare, accounting for 3% of the cases.

Clinically, metastatic lesions in the breast present as painless swellings with rapid growth [1,4]. Unlike primary tumours, the skin is not involved and the axillary node involvement is variable [1,4].

Mammogram shows well-circumscribed lesions, which lack microcalcifications [1,4]. The course of renal cell carcinoma is extremely variable and unpredictable both in its clinical development and metastasis [1]. Most patients with renal cell carcinoma following nephrectomy develop multiple metastases and die within 10 years [2].

A preceding history of extramammary carcinoma can be helpful in suspecting a mass as a possible metastasis, as radical surgery can be avoided and appropriate chemotherapy or radiation therapy can be ensured [3,4].

## Conclusion

A treatment strategy for metastatic breast diseases is based on a proper assessment of such cases by surgeons, radiologists and histopathologists.

## Competing interests

The author(s) declare that they have no competing interests.

## Authors' contributions

**AA **performed literature review, drafted and revised manuscript

**AV **evaluated histopathological features, edited and revised the manuscript

**HM **evaluated radiological images

**MS **contributed to the conception of the report

**NS **contributed to collection of the clinical data

All authors read and approved the final manuscript.
